# Screening for tuberculosis and prediction of disease in Portuguese healthcare workers

**DOI:** 10.1186/1745-6673-6-19

**Published:** 2011-06-09

**Authors:** José Torres Costa, Rui Silva, Felix C Ringshausen, Albert Nienhaus

**Affiliations:** 1 Hospital São João, EPE Alameda Professor Hernâni Monteiro, Porto, Portugal; 2 Faculty of Medicine, Porto University Alameda Professor Hernâni Monteiro, Porto, Portugal; 3 University Medical Centre Hamburg-Eppendorf, Institute for Health Services Research in Dermatology and Nursing, Hamburg, Germany

## Abstract

**Introduction:**

Results of systematic screening of healthcare workers (HCWs) for tuberculosis (TB) with the tuberculin skin test (TST) and interferon-γ release assays (IGRA) in a Portuguese hospital from 2007 to 2010 are reported.

**Methods:**

All HCWs are offered screening for TB. Screening is repeated depending on risk assessment. TST and QuantiFERON Gold In-Tube (QFT) are used simultaneously. X-ray is performed when TST is > 10 mm, IGRA is positive or typical symptoms exist.

**Results:**

The cohort comprises 2,889 HCWs. TST and IGRA were positive in 29.5%, TST-positive but IGRA-negative results were apparent in 43.4%. Active TB was diagnosed in twelve HCWs - eight cases were detected during screening and four cases were predicted by IGRA as well as by TST. However, the progression rate in IGRA-positive was higher than in TST-positive HCWs (0.4% vs. 0.2%, p-value 0.06).

**Conclusions:**

The TB burden in this cohort was high (129.8 per 100,000 HCWs). However, the progression to active TB after a positive TST or positive IGRA was considerably lower than that reported in literature for close contacts in low-incidence countries. This may indicate that old LTBI prevails in these HCWs.

## Introduction

Screening healthcare workers (HCWs) for latent tuberculosis infection (LTBI) and active tuberculosis (TB) disease is fundamental in infection control programmes in hospitals [1]. The tuberculin skin test (TST) was the first method available for detecting LTBI. However, the TST has known limitations, including cross-reactivity with bacillus Calmette-Guérin (BCG) and non-tuberculous mycobacteria (NTM) infections [2]. Recently, new *in vitro *assays that measure interferon (IFN)-γ released by sensitised T cells after stimulation with *Mycobacterium tuberculosis *antigens have been developed. These tests are more specific than the TST since they use antigens not shared by any of the BCG vaccine strains nor by the more common species of NTM [3]. Interferon-γ release assays (IGRAs) also have the advantage of correlating better with surrogate measures of exposure to *M. tuberculosis *[4-6] and have a higher predictive value for LTBI progression to active TB disease in close contact in low-incidence settings [7,8].

This paper represents a recent analysis of the growing cohort of HCWs tested with IGRA at the University Hospital of Porto, Portugal, between January 2007 and December 2010. In previous papers, TST and IGRA results were compared with respect to risk factors for LTBI [9,10]. Now the sample size has been increased from 1,218 to 2,884 HCWs for whom a direct comparison of TST and IGRA is possible. Furthermore, more than three years have passed since the introduction of IGRA screening at the hospital and the predictive value of TST and IGRA for developing active TB in HCWs can be analysed.

## Methods

All workers at the Hospital São João are offered TB screening following the Centers for Disease Control and Prevention (CDC) guidelines [1]. Upon commencement of employment, all workers are examined to exclude active TB disease and to assess the pre-employment status. Depending on the risk assessment, the examination is repeated annually or every other year. HCWs with close patient contacts in the infection and TB wards are considered to be at a high risk, workers with regular patient contacts in the other wards are considered to be at a medium risk and workers with no regular patient contacts and no contact with biological material are considered to be at a low risk. After unprotected contact with an infectious patient, co-worker or material, screening is performed as well.

Since January 2007, screening has been performed using TST and IGRA. A chest X-ray is performed in order to exclude active pulmonary disease when TST is considered positive (≥ 10 mm), when IGRA is positive and in HCWs with symptoms. BCG vaccination is assessed through the individual vaccination register. If no register is available, vaccination status is verified by scars. BCG vaccination for newborns is mandatory in Portugal and, until January 2000, was repeated depending on risk assessment and TST diameter. Therefore every HCW is considered to have been vaccinated at least once.

TST is performed by trained personnel following standard procedures. In brief, 0.1 mL (2 TU) of purified protein derivative (PPD, RT23; Statens Serum Institute, Copenhagen, Denmark) is injected. The TST is administered to the volar side of the forearm of the participants and read 72 to 96 hours after the application. The transverse diameter of the induration is measured by experienced personnel.

Before the TST application, an interview is performed and blood for the IGRA is drawn. For the IGRA, the QuantiFERON-TB^® ^Gold In-Tube Assay (Cellestis Limited, Carnegie, Australia) is used. This whole-blood assay uses overlapping peptides corresponding to ESAT-6, CFP-10 and a portion of the tuberculosis antigen TB7.7 (Rv2654). Stimulation of the antigenic mixture occurs within the tube used to collect blood. Tubes were incubated at 37 C overnight before centrifugation, and INF-γ release is measured by ELISA following the protocol of the manufacturer. All assays performed met the manufacturer's quality control standards. The test is considered positive when INF-γ is ≥ 0.35 IU after correction for the negative control. Observers were blinded to the results of the TST results.

A chi-square test was used to compare the frequencies of test results among different groups of participants. For risk factors assessed by ordinal variables, the proportions of positive test results were compared using the chi-square test of trend. A binomial test was used for the comparison of active TB rates and TB predicted rates of TST and IGRA. P < 0.05 was considered to be statistically significant. Adjusted odds ratios (OR) and 95% confidence intervals (CI) were calculated for different putative predictive variables using conditional logistic regression.

Data analysis was performed with SPSS, Version 14 (SPSS Inc., Chicago, Illinois). All persons gave their informed consent prior to their inclusion in the study. No additional data was collected for the study purpose only and analysis was performed with anonymous data. Therefore no endorsement by an ethics committee was required.

## Results

**Figure 1:**
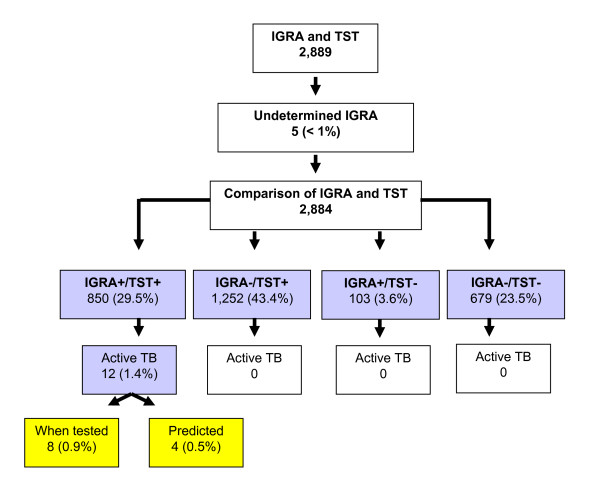
**Flow chart of study population**.

The flow chart of the study sample is given in Figure 1. Undetermined results of the IGRA were observed in 5 HCWs (5/2,889). A total of 850 HCWs (29.5%) was positive in TST and IGRA. Twelve of these HCWs (1.5%) were diagnosed with active TB. Four HCWs were diagnosed with TB more than three months after the positive TST and IGRA. The characteristics of the study population are given in Table 1. The cohort is predominantly female (71.7%) and the majority were repeatedly vaccinated with BCG (68.2%). Infection risk was considered moderate for 59.7% of the cohort. The mean follow-up time for the HCWs was 19 months, SD 5.2 month (no table).

**Table 1:** Study population for comparison of IGRA with TST

Age	N	%
< 25 years	301	10.4

25-29 years	821	28.5

30-39 years	791	27.4

40-49 years	534	18.5

≥ 50 years	437	15.2

Gender		

Female	2,068	71.7

Male	816	28.3

BCG vaccination		

Only at birth	917	31.8

One additional	1,022	35.4

Two additional	663	23.0

≥ 3 additional	282	9.8

Profession		

Administrator	388	13.5

Auxiliaries, cleaning staff	487	16.9

Technicians (radiology, lab, etc.)	170	5.9

Nurses	1,204	41.7

Physicians	635	22.0

Risk assessment		

Low risk	206	7.1

Moderate risk	1,722	59.7

High risk	956	33.1

Years working in healthcare		

Start of work	400	13.9

< 1 years	141	4.9

1-5 years	787	27.3

>5-10 years	465	16.1

10-20 years	573	19.9

≥ 20 years	518	18.0

IGRA was positive in 33% and TST was 10 mm or higher in 72.9% of the HCWs (Table 2). The probability of a positive IGRA increased with diameter in TST. However, 42.4% of the HCWs with a TST of ≥ 20 mm were negative in IGRA and 11.2% with a TST of < 5 mm were positive in IGRA.

**Table 2:** TST diameter by IGRA results

	IGRA					
**TST**	**Negative**		**Positive**		**Total**	
	
	**N**	**Row%**	**N**	**Row%**	**N**	**Col%**

0-4 mm	596	88.8	75	11.2	671	23.3

5-9 mm	83	74.8	28	25.2	111	3.8

10-14 mm	623	73.3	227	26.7	850	29.5

15-19 mm	468	53.7	404	46.3	872	30.2

≥ 20 mm	161	42.4	219	57.6	380	13.2

All	1,931	67.0	953	33.0	2,884	100.0

TST = Tuberculin skin test

IGRA = Interferon-γ release assay

Row% = % within the TST category

Col% - Column% = % of total falling into a certain TST category

P-value for linear trend < 0.001

Age is a risk factor for a positive TST (≥ 10 mm) as well as a positive IGRA (Table 3). However, the adjusted OR for the IGRA show a more pronounced dose-response relationship with age than the TST, e.g. OR in HCWs aged 50 or older for positive TST 2.0 and for positive IGRA 2.7 (Table 3). Gender was not associated with TST or IGRA. Repeated BCG vaccination did not influence TST, but was associated with a decreased probability of positive IGRA, e.g. OR = 0.4 (95% CI 0.3-0.6) for three or more additional vaccinations. Profession and risk assessment were not associated with TST or IGRA results. The number of years working in healthcare increased the probability of positive TST and IGRA. The highest OR (2.5) was observed for TST when working for 20 or more years in healthcare. However, due to high correlation between the variables of age and years working in healthcare, it is not possible to separate both effects.

**Table 3:** Adjusted odds ratios (OR) and 95% confidence intervals (CI) for tuberculin skin tests (TST) of ≥ 10 mm and positive interferon-γ release assays (IGRA)

	TST ≥ 10 mm			IGRA-positive		
**Age**	N (%)	OR	95%CI	N (%)	OR	95%CI

< 25 years	223 (74.1)	1	--	57 (18.9)	1	--

25-29 years	532 (64.8)	0.6	0.5-0.8	204 (24.8)	1.3	0.93-1.8

30-39 years	553 (69.9)	0.8	0.6-1.1	266 (33.6)	1.9	1.4-2.6

40-49 years	424 (79.4)	1.5	1.0-2.1	219 (41.0)	2.3	1.6-3.3

≥ 50 years	370 (84.7)	2.0	1.4-3.0	207 (47.4)	2.7	1.9-3.9

**Gender**						

Female	1,487 (71.9)	1	--	658 (31.8)	1	--

Male	615 (75.4)	1.2	0.97-1.4	295 (36.2)	1.1	0.9-1.3

**BCG vaccination**						

Only at birth	700 (76.3)	1	--	406 (44.3)	1	--

One additional	741 (72.5)	1.0	0.8-1.2	330 (32.3)	0.7	0.6-0.9

Two additional	460 (69.4)	1.0	0.7-1.2	165 (24.9)	0.6	0.5-0.7

3-10 additional	201 (71.3)	1.0	0.8-1.4	52 (18.4)	0.4	0.3-0.6

**Profession**						

Administrator	302 (77.8)	1		156 (40.2)	1	--

Auxiliaries, cleaning staff	335 (68.8)	0.8	0.6-1.1	180 (37.0)	1.0	0.7-1.3

Technicians (radiology, lab, etc.)	137 (80.6)	1.6	1.0-2.5	54 (31.8)	1.0	0.7-1.5

Nurses	864 (71.8)	1.3	0.95-1.8	317 (26.3)	0.8	0.6-1.1

Physicians	464 (73.1)	1.2	0.9-1.7	246 (38.7)	1.4	1.04-1.9

**Risk assessment**						

Low risk	176 (85.4)	1	--	85 (41.3)	1	--

Moderate risk	1,289 (74.9)	0.6	0.4-0.91	549 (31.9)	0.8	0.6-1.2

High risk	637 (66.6)	0.4	0.3-0.6	319 (33.4)	0.9	0.7-1.3

Years working in healthcare*						

Start of work	289 (72.3)	1	--	112 (28.0)	1	--

< 1 years	84 (59.6)	0.6	0.4-0.92	26 (18.4)	0.7	0.4-1.1

1-5 years	502 (63.8)	0.7	0.5-0.91	211 (26.8)	1.1	0.8-1.4

> 5-10 years	324 (69.7)	0.9	0.7-1.2	154 (33.1)	1.4	1.06-2.0

10-20 years	454 (79.2)	1.5	1.1-2.1	212 (37.0)	1.6	1.2-2.2

≥ 20 years	449 (86.7)	2.5	1.8-3.6	238 (45.9)	1.9	1.4-2.5

* Correlation between age and years working in healthcare r = 0.82. Separate models were calculated for these variables

Discordant TST+/IGRA- combinations are most likely (57.8%) in HCWs younger than 25 years and less likely in HCWs older than 50 years (40%). Gender is not associated with discordant TST and IGRA results (Table 4). The probability of TST+/IGRA- discordance increased from 35.8% in those with BCG vaccination at birth only to 55.7% in those with three or more repeated vaccinations. Technicians (52.4%) and nurses (48.6%) had the highest rates of TST+/IGRA- discordant results.

**Table 4:** Concordant and discordant tuberculin skin test (TST) of ≥ 10 mm and interferon-γ release assay (IGRA) results depending on putative risk factors

	TST/IGRA								
	**Neg./Neg.**		**Pos./Neg.**		**Neg./Pos.**		**Pos./Pos.**		**P**

**Age**	N	%	n	%	n	%	n	%	<0.001
	
< 25 years	70	23.3	174	57.8	8	2.7	49	16.3	
	
25-29 years	258	31.4	359	43.7	31	3.8	173	21.1	
	
30-39 years	203	25.7	322	40.7	35	4.4	231	29.2	
	
40-49 years	93	17.4	222	41.6	17	3.2	202	37.8	
	
≥ 50 years	55	12.6	175	40.0	12	2.7	195	44.6	

**Gender**									

Female	508	24.6	902	43.6	73	3.5	585	28.3	

Male	171	21.0	350	42.9	30	3.7	265	32.5	0.078

**BCG vacci.**									

At birth	183	20.0	328	35.8	34	3.7	372	40.6	

Plus one additional	245	24.0	447	43.7	36	3.5	294	28.8	

Plus two	178	26.8	320	48.3	25	3.8	140	21.1	

Plus 3-10	73	25.9	157	55.7	8	2.8	44	15.6	<0.001

**Profession**									

Administrator	72	18.6	160	41.2	14	3.6	142	36.6	

Auxiliaries, cleaning	133	27.3	174	35.7	19	3.9	161	33.1	

Technicians (radiology, lab, etc.)	27	15.9	89	52.4	6	3.5	48	28.2	

Nurses	302	25.1	585	48.6	38	3.2	279	23.2	

Physicians	145	22.8	244	38.4	26	4.1	220	34.6	<0.001

**Risk**									

Low	27	13.1	94	45.6	3	1.5	82	39.8	

Moderate	378	22.0	795	46.2	55	3.2	494	28.7	

High	274	28.7	363	38.0	45	4.7	274	28.7	<0.001

**Years in healthcare**									

Start of work	96	24.0	192	48.0	15	3.8	97	24.3	

< 1 years	51	36.2	64	45.4	6	4.3	20	14.2	

1-5 years	248	31.5	328	41.7	37	4.7	174	22.1	

> 5-10 years	124	26.7	187	40.2	17	3.7	137	29.5	

10-20 years	103	18.0	258	45.0	16	2.8	196	34.2	

≥ 20 years	57	11.0	223	43.1	12	2.3	226	43.6	<0.001

Fifty-seven HCWs (2.0%) had a history of active TB disease since 2005 and were treated accordingly. Of these, 86% had a TST of ≥ 10 mm and 59.6% a positive IGRA when screened in the scope of this study (Table 5). Eight HCWs had active TB at the time of screening, and progression to active TB within 4 to 24 months of screening occurred in four HCWs. Sensitivity for prevalent and for predicted active TB was 100% for both TST of ≥ 10 mm and IGRA. The rate of prevalent and predicted active TB in IGRA-positive HCWs (0.8% and 0.4%) was twice as high as the rates for HCWs with TST of ≥ 10 mm (0.4% and 0.2%). However, the difference was only statistically significant (p = 0.008) for prevalent active TB. The p-value for the different progression rates was 0.06 (Table 5).

**Table 5:** Old, active, and predicted TB by TST and IGRA results

	TST				IGRA				Total	
		
	0-9 mm		≥ 10 mm		Neg.		Pos.			
TB history	n	%	n	%	n	%	N	%	n	%*

Old TB	8	14.0	49	86.0	23	40.4	34	59.6	57	2.0

Prevalent active TB	0	--	8	0.4*	0	--	8	0.8*	8	0.3

Predicted TB**	0	--	4	0.2*	0	--	4	0.4*	4	0.1

No TB	774	27.5	2102	72.9	1908	67.8	953	32.8	2815	97.6

* Column%

** Time between test and diagnosis of active TB > 3 months

Progression rate:

TST ≥ 10 mm = 0.2%

IGRA positive = 0.4%, p = 0.06

Active TB rate:

TST ≥ 10 mm = 0.4%

IGRA positive = 0.8%, p = 0.008

## Discussion

This is the first study to report sensitivity for disease progression in HCWs simultaneously tested with IGRA and TST. However, even though the study sample was huge (n = 2,884), calculation of the disease progression rate is based on four cases only. In IGRA-positive HCWs, it was twice as high as in HCWs with TST of ≥ 10 mm. However, the difference was not statistically significant. The progression rate we observed was considerably lower than the one observed in close contacts in a German cohort [7,8]. Apart from a shorter follow-up period in our study, this indicates that old infections, which have a lower progression risk, prevail in the HCW cohort. As with TST, IGRAs are not able to distinguish old from recent LTBI [11]. In HCWs with a positive IGRA, the likelihood of a recent LTBI can only be assessed by evaluation of the exposure situation within the last months before the IGRA is performed. This should be done from case to case, as the exposure assessment following CDC [1] was not helpful in this endeavour.

Even though the sample size increased from 1,219 to 2,884 compared to our first publication [9], the rates for positive IGRA and TST of ≥ 10 mm are very similar (32.6% versus 33.1% for IGRA and 74.2% versus 72.9% for TST of ≥ 10 mm). The rate of positive IGRA is considerably higher in Portuguese HCWs than in HCWs from other West-European countries, reflecting the higher TB-incidence in Portugal than in other West-European countries [12-14]. The twelve cases of active TB observed in our population gives rise to a TB incidence rate of 129.8 per 100,000 HCWs. The rate is considerably lower than the rate of 191.6 per 100,000 HCWs observed in our previous publication [9]. But the rate is still about four times higher than the annual incidence rate in the general population in Portugal in 2006 of 32/100,000 [9]. This comparison illustrates the beneficial aspects of systematic screening of HCWs in a medium-incidence country and the increased risk of TB infection in HCWs.

In Portugal, BCG vaccination is universal and, until January 2000, was repeated when a person was negative in the TST. This makes it difficult to examine the influence of BCG vaccination on TST or IGRA. Little is currently known about the effect of repeated BCG vaccination on the probability of TST+/IGRA- discordant results [11,15]. As was expected, repeated BCG vaccination increased the probability of TST results higher than 10 mm. However, a recent BCG vaccination or a repeated BCG vaccination decreased the probability of a positive IGRA. This might indicate a protective effect of BCG vaccination or may have been caused by the revaccination schema: TST-negative HCWs are revaccinated, inducing a positive TST, without changing the IGRA. Increasing the cut-off for a positive TST from 10 to 15 mm reduced the number of positive TSTs (72.9 vs. 43.4), but also increased the probability of a positive IGRA not detected by TST (10.8% vs. 34.6%, calculated from Table 2). Therefore, this strategy is not suitable to reduce the specificity problems of the TST in a population which is BCG-vaccinated.

Nurses had a higher rate of TST+/QFT- results than physicians (48.6 vs. 38.6%, Table 4) but were also tested more often than physicians with TST [9]. This indicates that risk assessment may be biased by using TST. The reason why nurses underwent TST more often than doctors is unknown; but one reasonable assumption is that they were more compliant in earlier contact tracings.

Working in healthcare is a well-known risk factor for TB [16,17]. In our data, the probability of a positive IGRA (Table 3) or of TST+/IGRA+ concordance (Table 4) increased with the number of years spent in healthcare. However, this association might be explained by age alone. Surprisingly, neither risk assessment [1] nor profession was associated with TST or with IGRA, or the association observed was in an unexpected direction. In the two European fingerprint studies [18,19], the majority of work-related active TB cases occurred when the infection risk was considered to be low and preventive measures were not in place because TB was not suspected. Rotation of the staff is a further potential explanation for the lack of this association. Therefore risk assessment should be reconsidered.

Screening HCWs for LTBI with TST in our populations had severe shortcomings. The rate of TST of > 10 mm was high and was influenced by repeated BCG vaccination, allowing little discrimination between HCWs at risk of having or of progressing to active TB. On the contrary, the IGRA was not influenced by BCG vaccination. Therefore our data corroborates the conclusion of other HCW studies [20,21] that the TST is not useful in contact investigations among BCG-vaccinated HCWs, while IGRA may provide additional information for the diagnosis and strategic management of preventive treatment in BCG-vaccinated HCW.

All eight HCWs diagnosed with active TB were positive in both tests. In summary, the IGRA was therefore better than the TST in screening HCWs for LTBI and active TB. Overall, screening of HCWs was successful because of the high number of active TB cases identified through this systematic screening. Future studies should investigate the incidence of new TB infections and the beneficial effect of chemoprevention in these HCWs.

## Competing interests

The authors declare that they do not have any direct or indirect personal relationship, affiliation or association with any party with whom they deal in their day-to-day work that would give rise to any actual or perceived conflict of interest.

## Authors' contributions

JTC designed the study, performed the physical examinations, and was involved in drafting of the paper. RS was involved in data collection and drafting of the paper. FR was involved in data analysis and drafting of the paper. AN analysed the data and wrote the first draft of the paper. All authors read and approved the final manuscript.
